# Supplementary figures and images for: 4-Aminoquinolines block heme iron reactivity and interfere with artemisinin action
Source: eLife. 2026 Mar 23;14:RP108976. doi: 10.7554/eLife.108976 (PMC13008355; doi:10.7554/eLife.108976)

Figure 3- source data 5

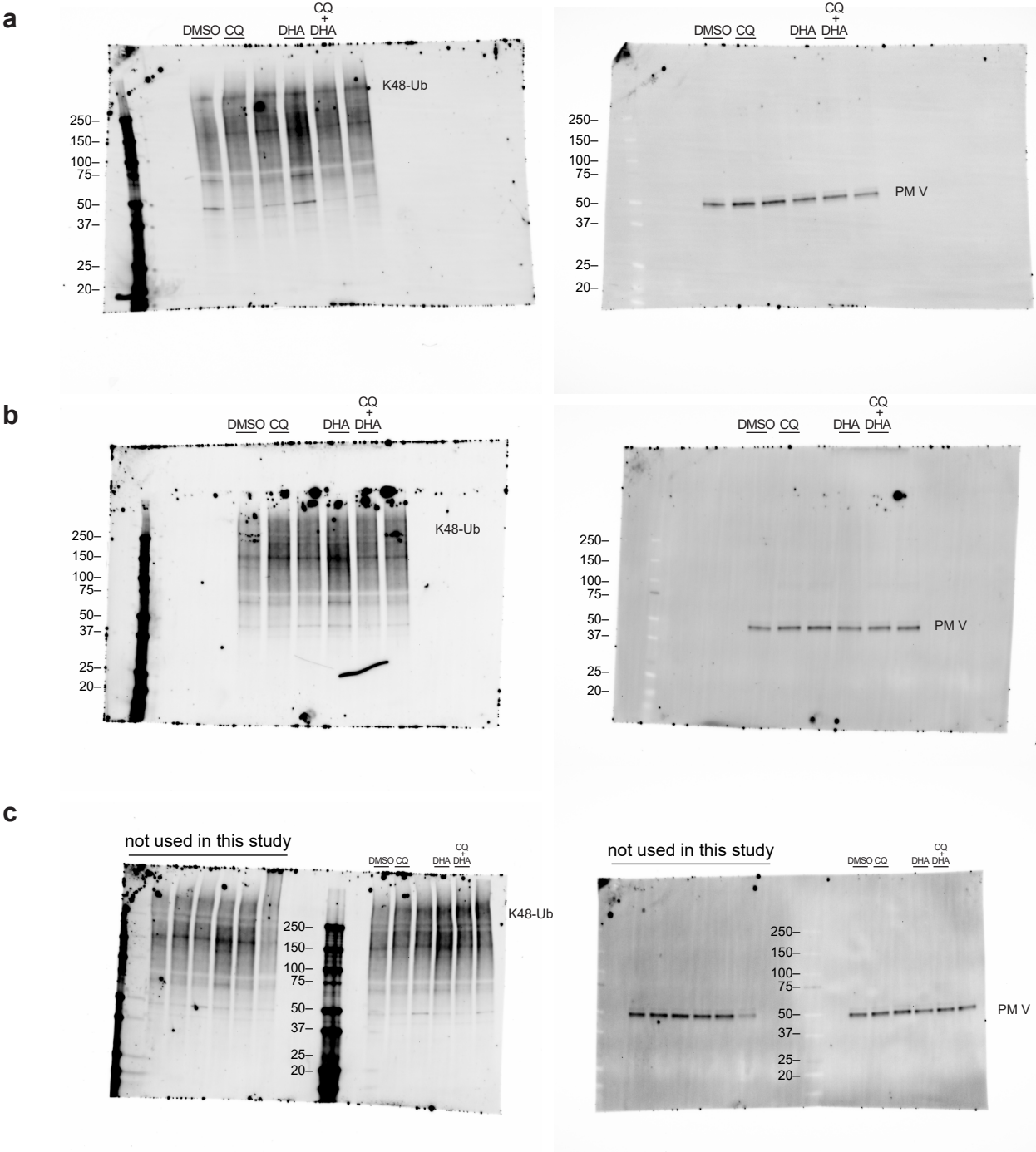

Supplement: Figure 3—source data 5. [file elife-108976-fig3-data5.zip › Figure 3- source data 5- Uncropped labled western blots/Figure 3- source data 5- Uncropped labled western blots.pdf]

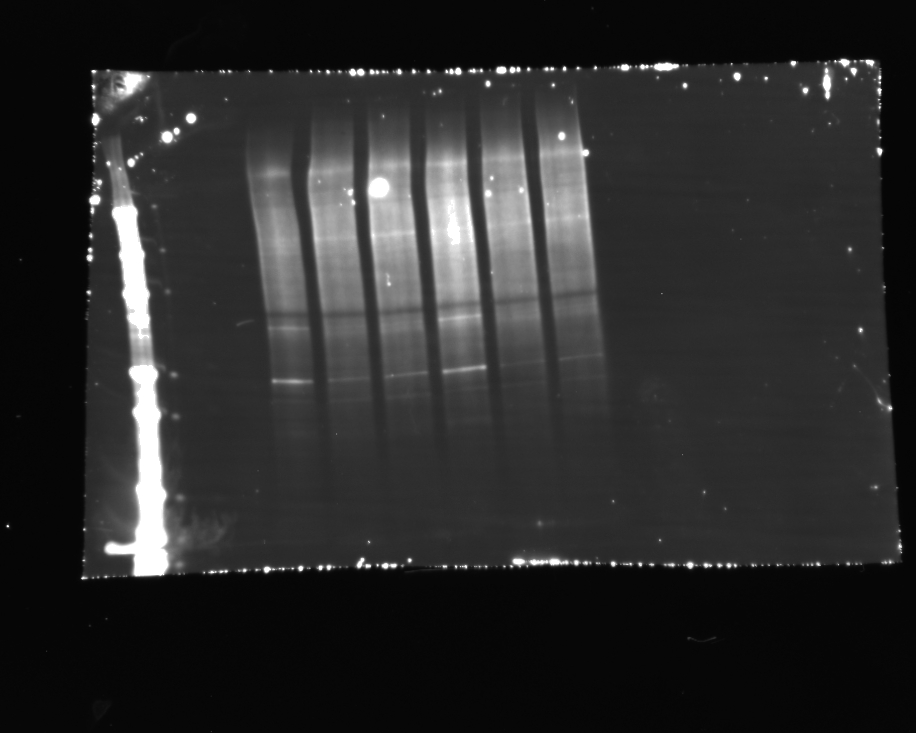

Supplement: Figure 3—source data 6. [file elife-108976-fig3-data6.zip › Figure 3- source data 6- Western blot raw images/Rep 1 (main figure)/K48/Melissa 2024-05-22 10h58m32s 120.000s(Alexa 680).raw16.tif]

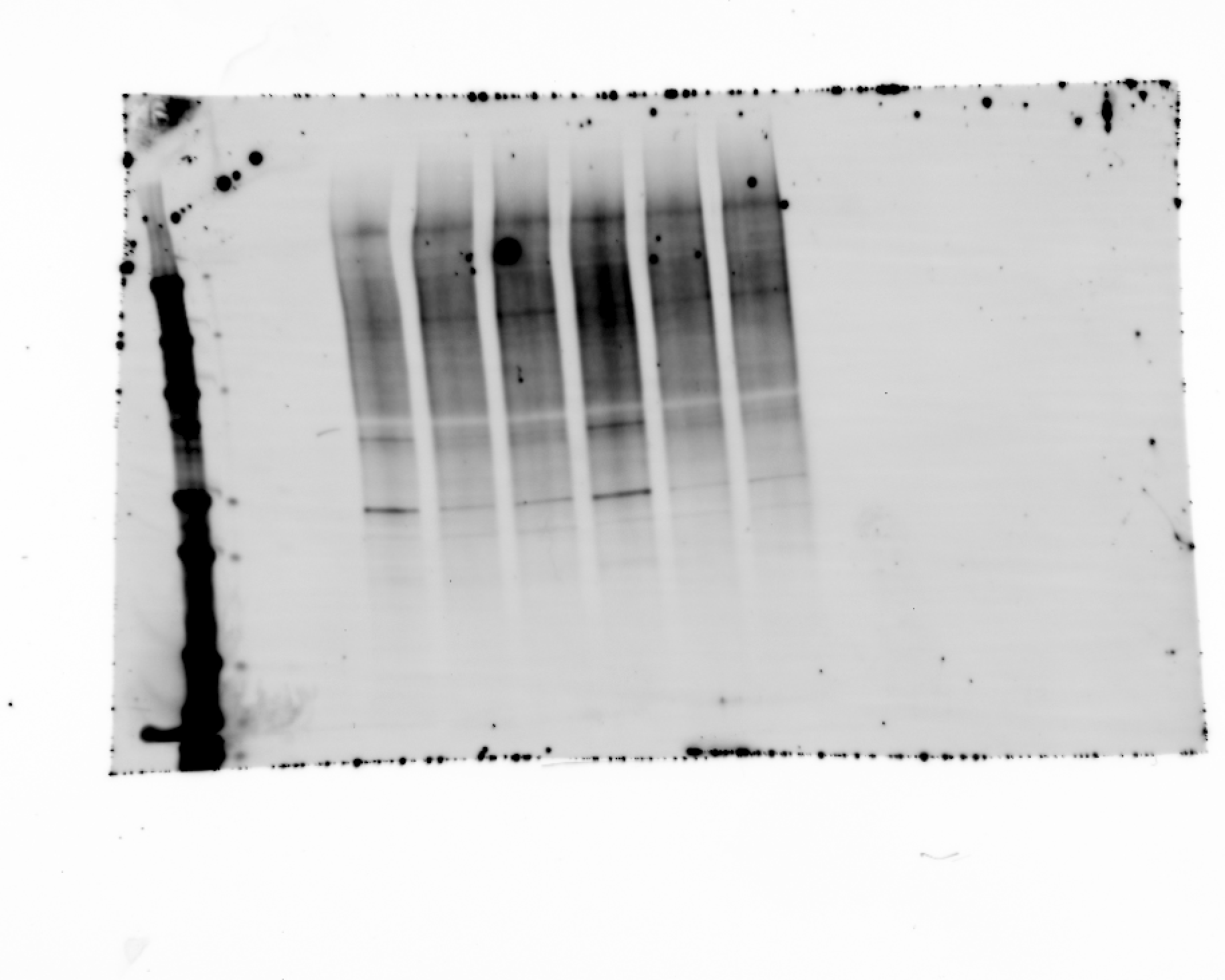

Supplement: Figure 3—source data 6. [file elife-108976-fig3-data6.zip › Figure 3- source data 6- Western blot raw images/Rep 1 (main figure)/K48/Melissa 2024-05-22 10h58m32s 120.000s(Alexa 680).tif]

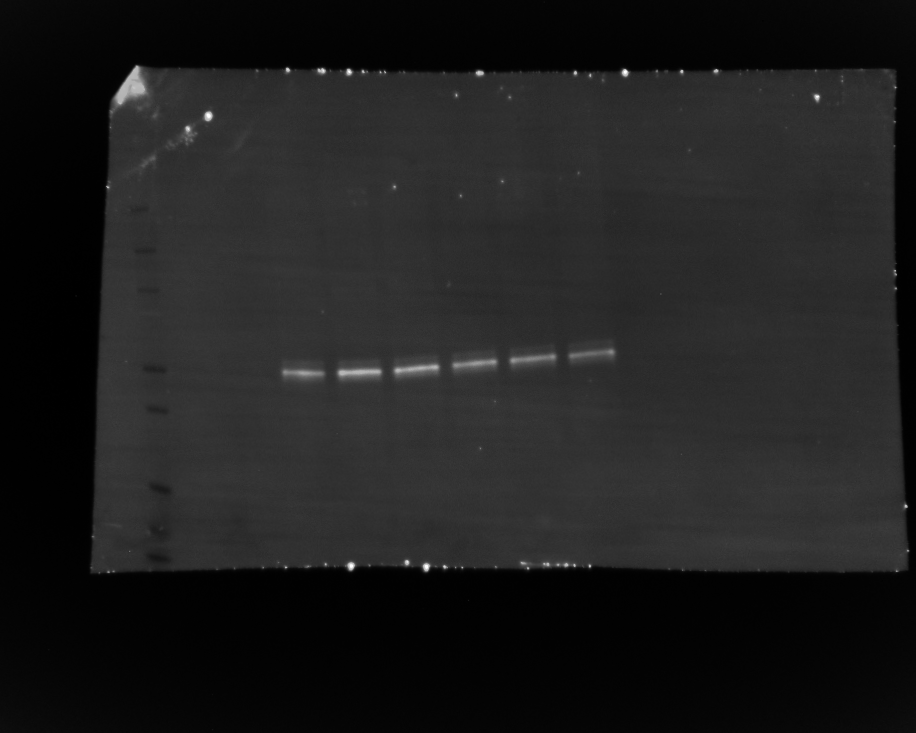

Supplement: Figure 3—source data 6. [file elife-108976-fig3-data6.zip › Figure 3- source data 6- Western blot raw images/Rep 1 (main figure)/PM V/Melissa 2024-05-24 10h28m45s 10.000s(IRDye 800CW).raw16.tif]

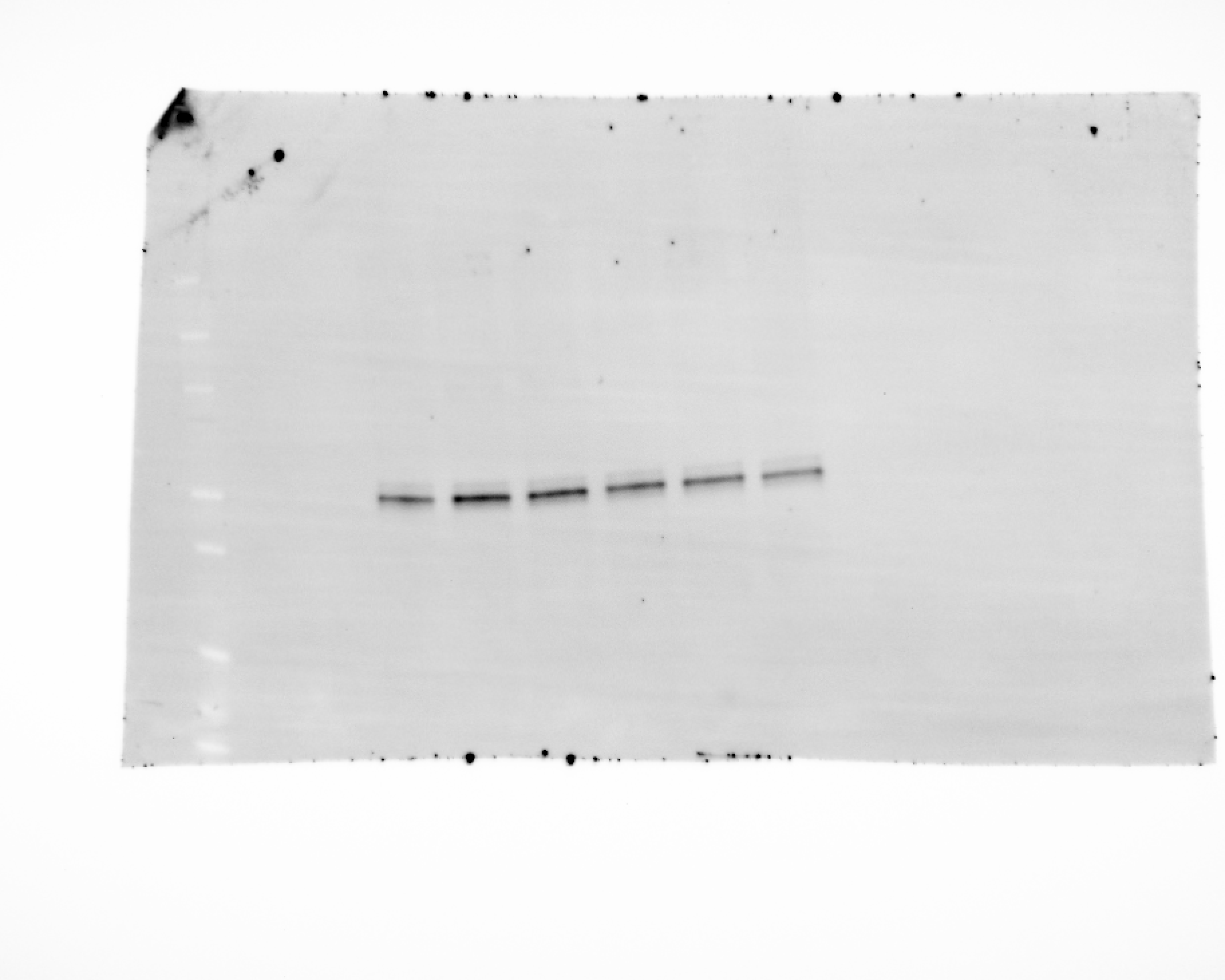

Supplement: Figure 3—source data 6. [file elife-108976-fig3-data6.zip › Figure 3- source data 6- Western blot raw images/Rep 1 (main figure)/PM V/Melissa 2024-05-24 10h28m45s 10.000s(IRDye 800CW).tif]

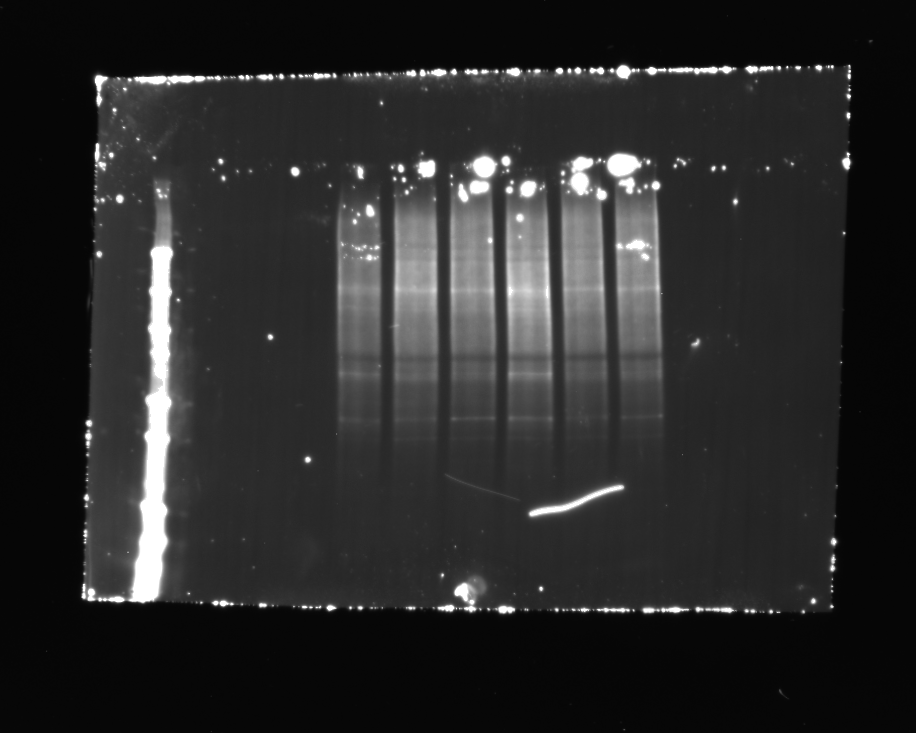

Supplement: Figure 3—source data 6. [file elife-108976-fig3-data6.zip › Figure 3- source data 6- Western blot raw images/Rep 2/K48/Melissa 2024-05-24 10h33m11s 120.000s(Alexa 680).raw16.tif]

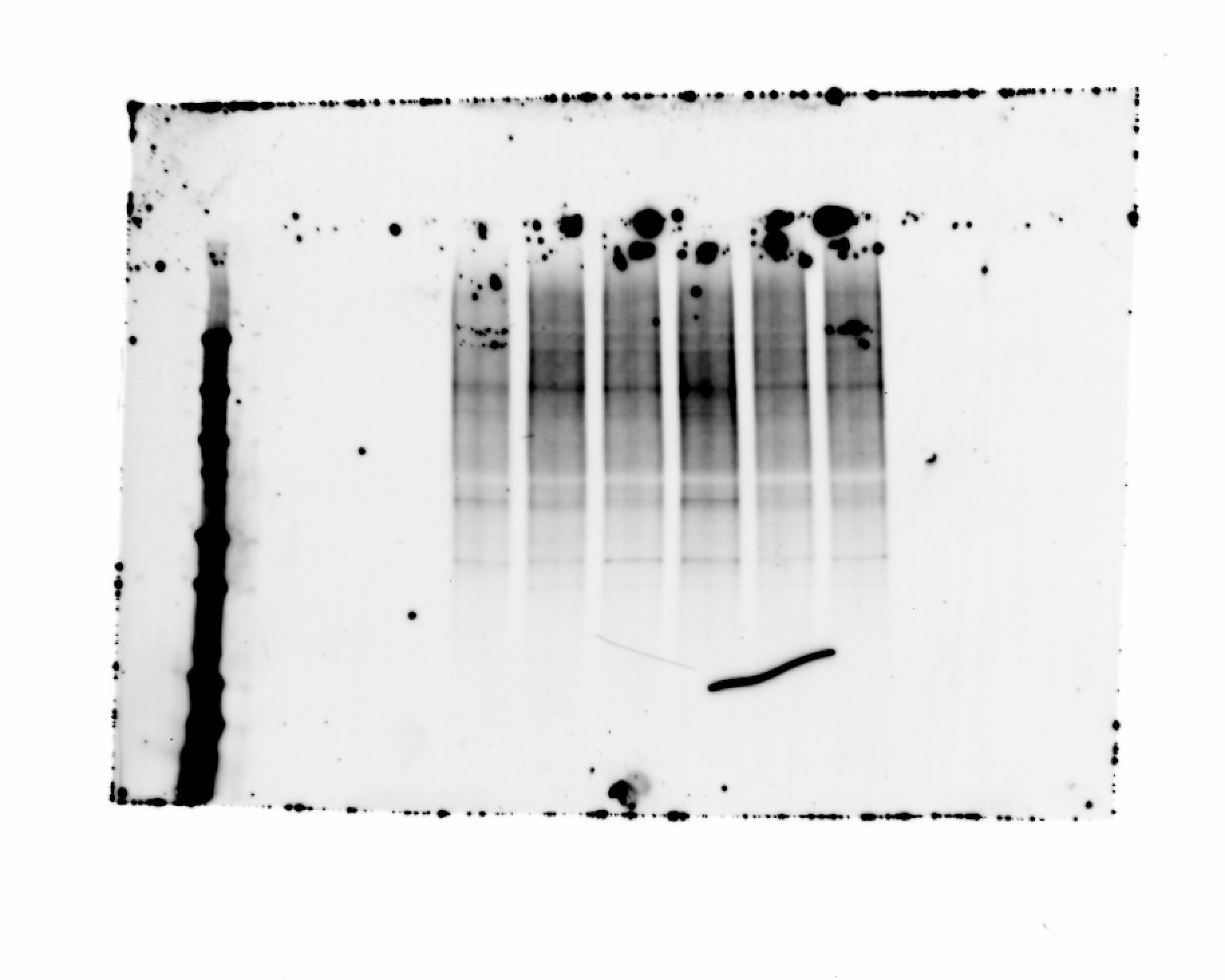

Supplement: Figure 3—source data 6. [file elife-108976-fig3-data6.zip › Figure 3- source data 6- Western blot raw images/Rep 2/K48/Melissa 2024-05-24 10h33m11s 120.000s(Alexa 680).tif]

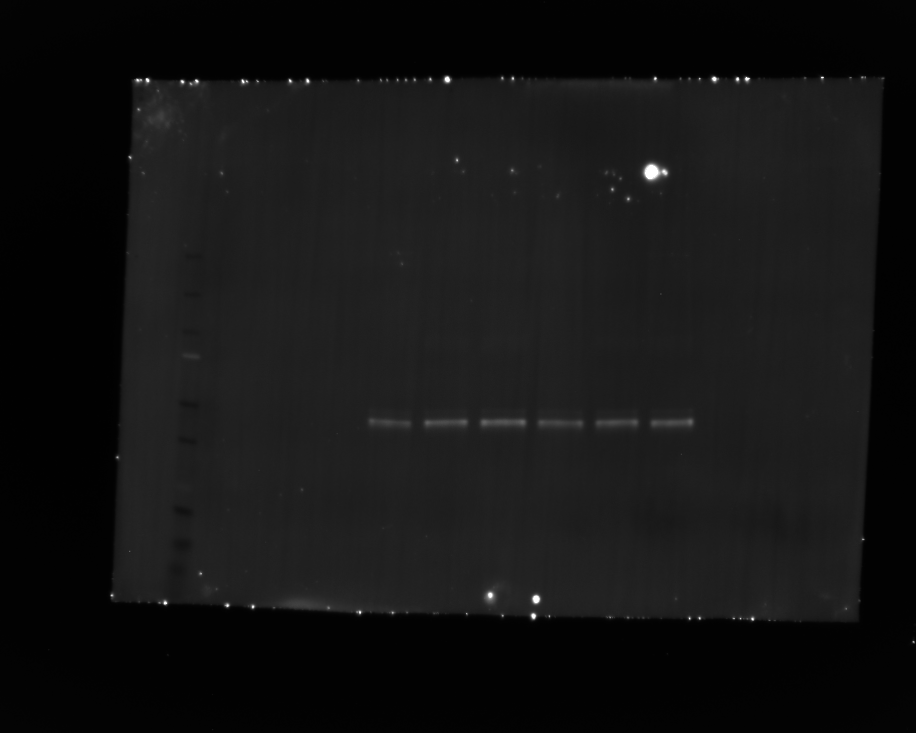

Supplement: Figure 3—source data 6. [file elife-108976-fig3-data6.zip › Figure 3- source data 6- Western blot raw images/Rep 2/PMV/Melissa 2024-05-29 13h17m41s 45.000s(IRDye 800CW).raw16.tif]

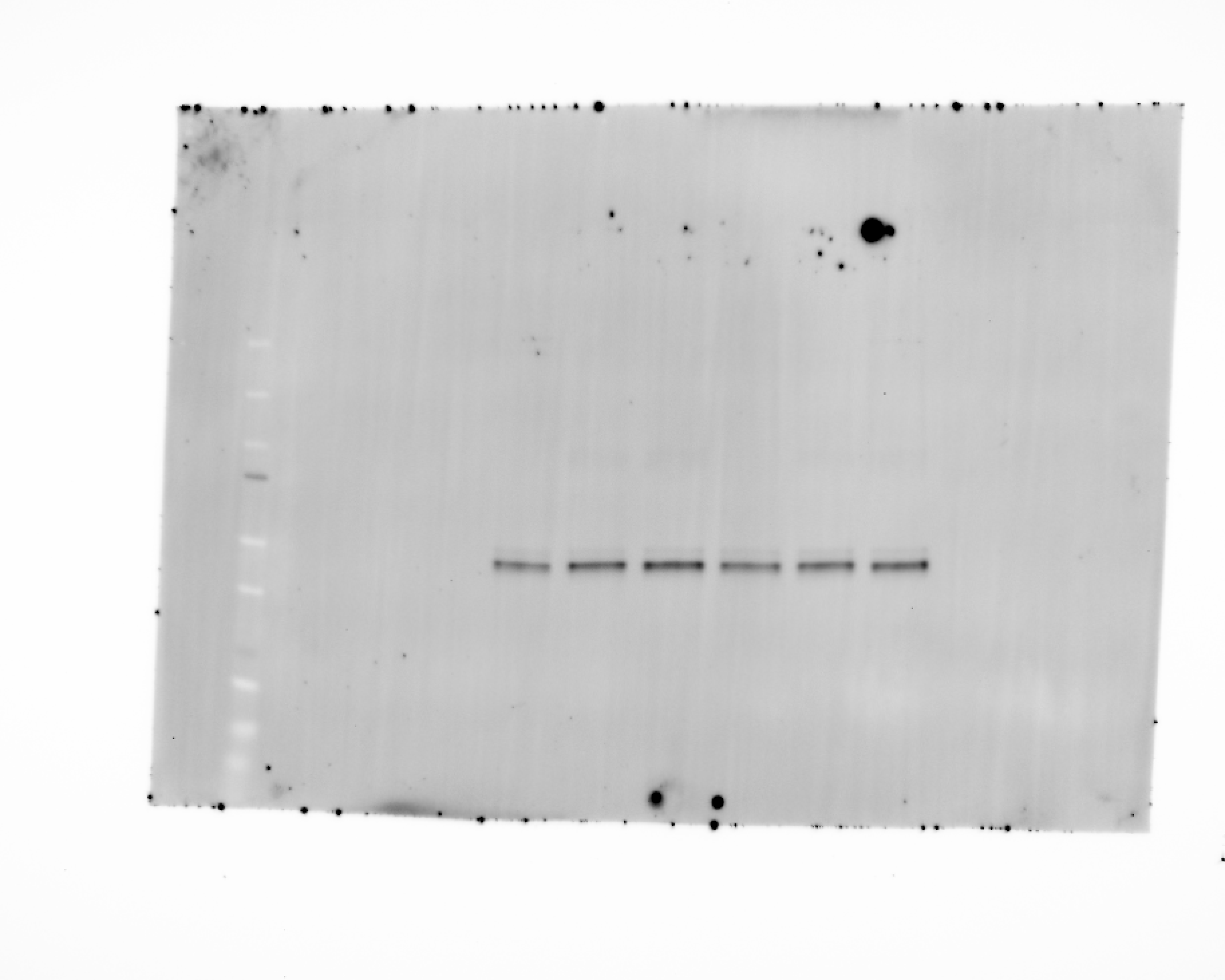

Supplement: Figure 3—source data 6. [file elife-108976-fig3-data6.zip › Figure 3- source data 6- Western blot raw images/Rep 2/PMV/Melissa 2024-05-29 13h17m41s 45.000s(IRDye 800CW).tif]

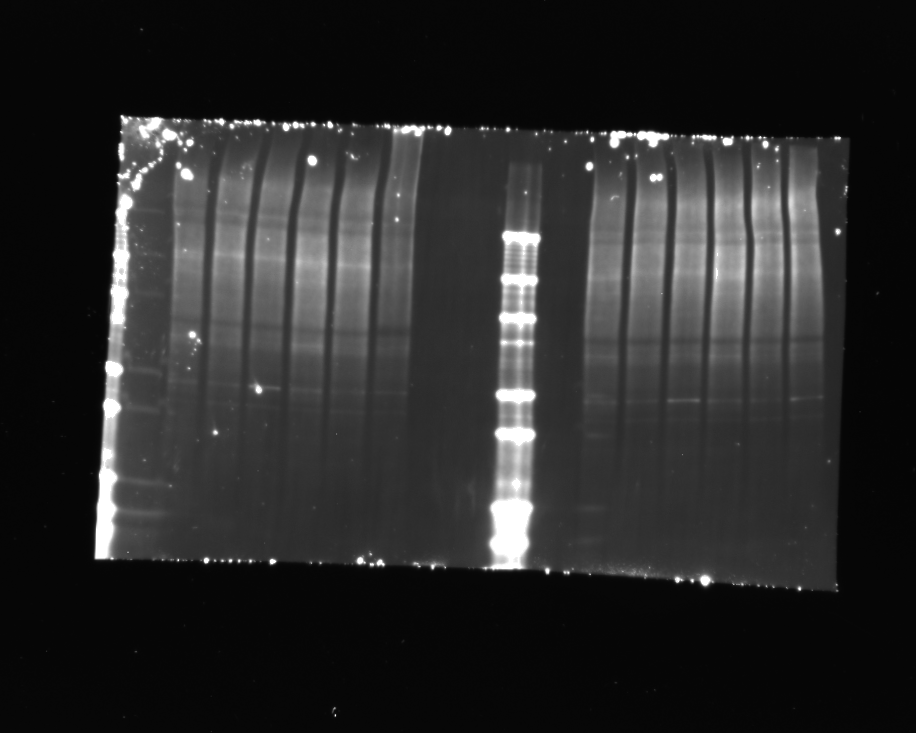

Supplement: Figure 3—source data 6. [file elife-108976-fig3-data6.zip › Figure 3- source data 6- Western blot raw images/Rep 3/K48/Melissa 2024-06-07 15h38m04s 120.000s(Alexa 680).raw16.tif]

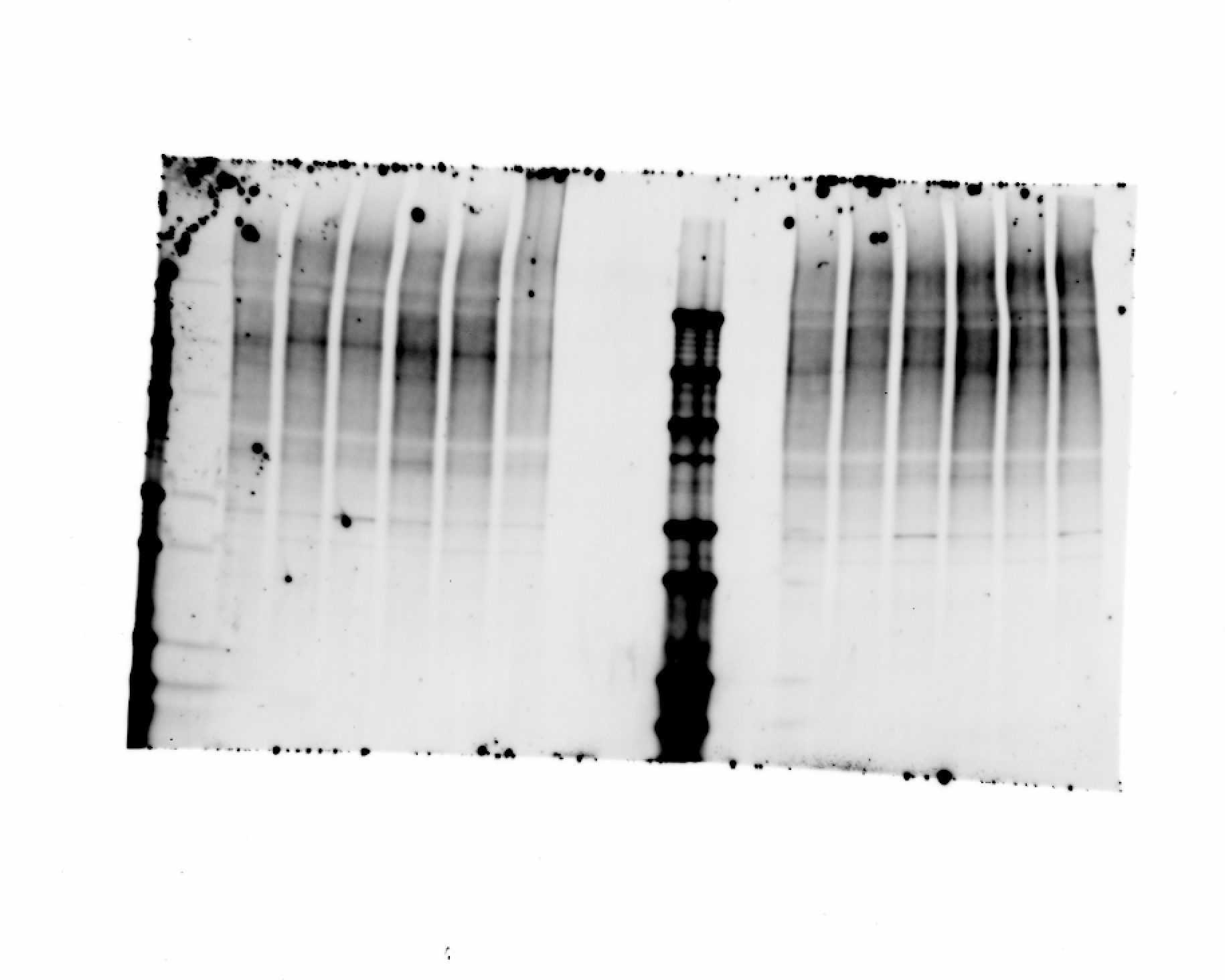

Supplement: Figure 3—source data 6. [file elife-108976-fig3-data6.zip › Figure 3- source data 6- Western blot raw images/Rep 3/K48/Melissa 2024-06-07 15h38m04s 120.000s(Alexa 680).tif]

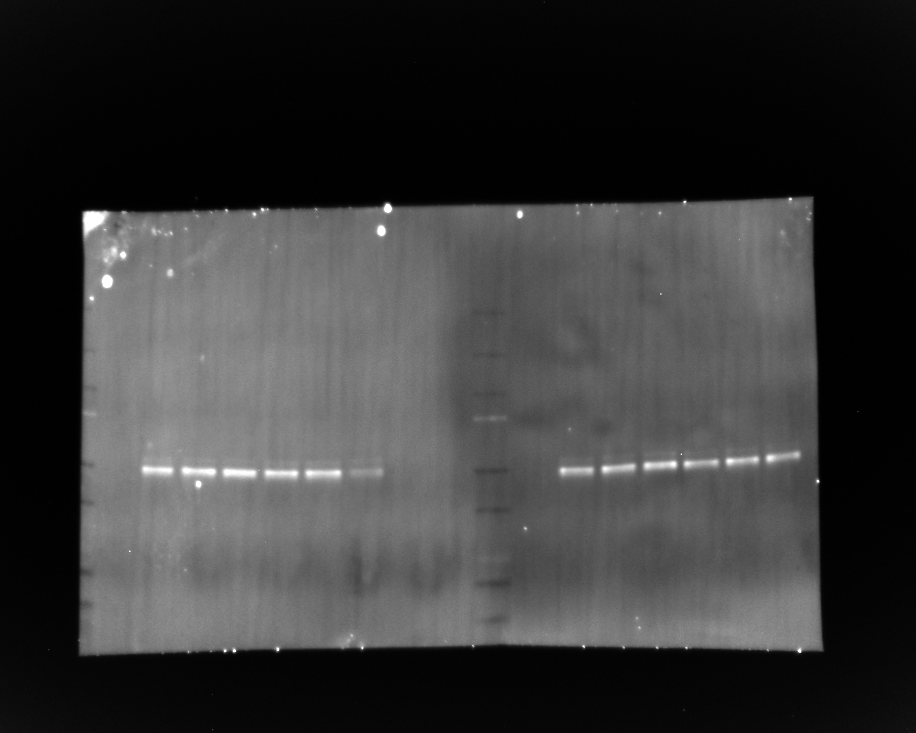

Supplement: Figure 3—source data 6. [file elife-108976-fig3-data6.zip › Figure 3- source data 6- Western blot raw images/Rep 3/PM V/Melissa 2024-06-10 13h48m19s 30.000s(IRDye 800CW).raw16.tif]

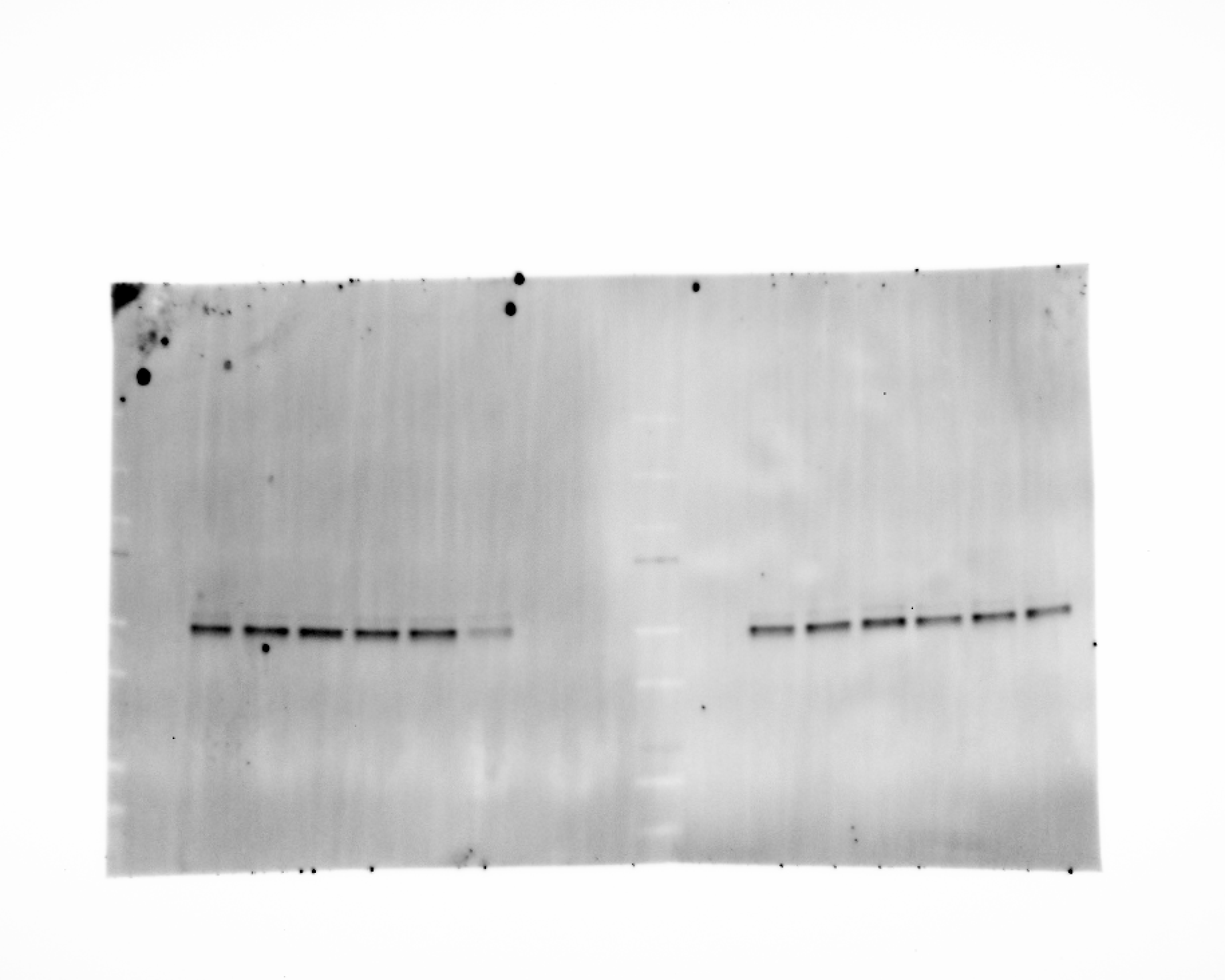

Supplement: Figure 3—source data 6. [file elife-108976-fig3-data6.zip › Figure 3- source data 6- Western blot raw images/Rep 3/PM V/Melissa 2024-06-10 13h48m19s 30.000s(IRDye 800CW).tif]
